# Pre-, pro- and synbiotics in cancer prevention and treatment—a review of basic and clinical research

**DOI:** 10.3332/ecancer.2018.869

**Published:** 2018-09-05

**Authors:** Alasdair J Scott, Claire A Merrifield, Jessica A Younes, Elizabeth P Pekelharing

**Affiliations:** 1 Department of Surgery and Cancer, St Mary’s Hospital, Imperial College London, London, W2 1NY, UK; 2 Winclove Probiotics BV, 1032 LB Amsterdam, The Netherlands

**Keywords:** microbiome, cancer, oncology, probiotics, prebiotics, synbiotics

## Abstract

There is a growing appreciation of the role of the human microbiota in the pathophysiology of cancer. Pre-, pro- and synbiotics are some of the best evidenced means of manipulating the microbiota for therapeutic benefit and their potential role in the prevention and treatment of cancer has garnered significant interest. In this review, we discuss how these agents may have oncosuppressive effects by maintaining intestinal barrier function, immunomodulation, metabolism and preventing host cell proliferation. We highlight the epidemiological and trials-based evidence supporting a role for pre-, pro- and synbiotics in the prevention of cancer. Ultimately, there is more evidence in support of these agents as adjuncts in the treatment of cancer. We discuss their roles in optimising the efficacy and/or minimising the adverse effects of chemotherapy and radiotherapy, antibiotics and surgery. Although we see significant promise in the application of pre-, pro- and synbiotics for clinical benefit in oncology patients, the field is very much in its infancy and oncologists face substantial challenges in advising their patients appropriately.

## Introduction

It is becoming increasingly well-established that the mammalian microbiota can play a role in tumourigenesis, propagation and metastasis of tumour cells, and can affect both the efficacy and toxicity of cancer treatment. Pre-, pro- and synbiotics currently represent the principal therapies directed towards positive manipulation of the microbiota (compared to negative manipulation with antibiotics) and this review will explore their potential therapeutic applications with regards to cancer. The definitions of pre- and probiotic have undergone several revisions in recent years as our understanding of the science has changed. A prebiotic is defined as ‘a substrate that is selectively utilised by host microorganisms conferring a health benefit’ [1]*.* This includes a variety of substances; the best studied being the nondigestible oligosaccharides fructooligosaccharide (FOS, such as found in onion and garlic) and galactooligosaccharide. These agents exert their effects by promoting the growth and/or function of commensal microorganisms with properties beneficial to the host. In contrast, probiotics are ‘live microorganisms that, when administered in adequate amounts, confer a health benefit on the host’ [2]*.* Although many fermented foods (such as kimchi or kefir) contain live organisms, most are not considered probiotics, as it is the *foodstuff*, rather than the organisms themselves, that confers the health benefit and they often do not contain sufficient quantities of organisms to be considered a probiotic. Synbiotics are a combination of pre- and probiotics in a single formulation. Probiotics comprise several specific microorganisms, such as *Lactobacilli* or *Bifidobacteria* genera, which can be formulated as a single agent or multi-strain preparations. Probiotics are usually administered orally, either in a yoghurt or as freeze-dried live organisms ingested as a powder or in a capsule, and are design to survive passage to the lower gastrointestinal (GI) tract. Putative mechanisms of action by which probiotics exert beneficial effects are many, varied and often strain specific [2, 3]. They may mitigate the effects of pathogenic organisms on the host by competitive exclusion, direct antagonism, neutralisation of pathogenic bacterial toxins and maintenance of intestinal barrier function (reviewed by Oelschlaeger *et al* [3]). Probiotics have significant metabolic effects including vitamin biosynthesis, bile acid metabolism, production of short-chain fatty acids (SCFAs) and neutralisation of carcinogens [4–6]. Probiotics may modulate the host immunity towards an oncosuppressive phenotype [7]. Finally, strains may also modify intestinal motility, gas production and stool consistency [8]. It is important to note that the beneficial effects of probiotics are delivered during intestinal transit and do not require colonisation of the host *per se* [9]. Pre-clinical and clinical studies have investigated the use of pre-, pro- and synbiotics in numerous aspects pertinent to oncology patients. We will cover the broad anticancer mechanisms of pre-, pro- and synbiotics before discussing the evidence regarding their use in the prevention and treatment of cancer.

## Anticancer mechanisms of pre-, pro- and synbiotics

As reviewed in this special issue (Alexander *et al* [10]) and elsewhere [11, 12] there is a strong evidence that the human microbiome plays a role in carcinogenesis and can be viewed as a key partner in a triumvirate ‘interactome’ with the host and the environment. Given this pivotal role, there is a significant interest in how the modulation of the microbiome via pre-, pro- and synbiotics may aid in the prevention and treatment of cancer. In support of an anticarcinogenic effect of pre-, pro and synbiotics there are plausible mechanisms which can broadly be characterised into intestinal barrier function, immunomodulatory, metabolic and antiproliferative effects (see Dos Reis *et al* [13] and Commane *et al* [7] for more in-depth review).

### Intestinal barrier function

The physical separation of the microbiota from the host is a key tenet of their symbiosis. Interruption of the physical barriers between microbiota and the host is known to be a key driver of disease, including cancer [12]. In the intestine, this barrier comprises enterocytes and cell junction proteins (forming the ‘tight-junction’ between cells), immune cells and secretory IgA, goblet cells and mucus, Paneth cells and antimicrobial peptides. The relationship between barrier failure, carcinogenesis and inflammation is complex and interdependent but is exemplified by mucin 2 homozygous knockout mice which do not produce intestinal mucus to act as a barrier and spontaneously develop colorectal cancer (CRC) [75]. Patients with ulcerative colitis also have defective barrier function which contributes to their increased risk of CRC. Pre-clinical work in colonocyte cell-lines and animal models has demonstrated that probiotics upregulate epithelial tight junction formation and mucus secretion [14–17]. Studies in humans have also demonstrated the enhanced colonic epithelial integrity with probiotic administration [18, 19]. Furthermore, in a rodent model, a synbiotic cocktail upregulated genes associated with the tight-junction formation and mucus production and inhibited the development of CRC [20].

### Immunomodulation

The microbiota is essential for maturation of the immune system and priming an anticancer response, both in the gut and at distant sites. Dendritic cells, natural killer (NK) cells and T cells are the key effectors for the detection and deletion of damaged and potentially carcinogenic host cells [21]. Probiotics interact with dendritic cells via cell-surface pattern recognition receptors, such as toll-like receptors, which in turn provoke T cell and NK cell responses. Syn- and probiotic preparations containing *Lactobacillus casei* or* Bifidobacterium lactis* have been shown to enhance NK cell activity in both rodent models [22] and human studies [23, 24]. Rodent models have also shown that probiotic-induced NK cell activity can suppress tumour growth [25, 26]. In a particularly interesting example, Sivan *et al* [27] found that genetically similar mice housed by different suppliers exhibited differential growth kinetics of subcutaneously injected melanoma cells. Tumours grew significantly more rapidly in mice sourced from Taconic Farms (TAC) than those from Jackson Laboratory (JAX). However, oral gavage of live, but not heat attenuated, *Bifidobacterium spp.* into TAC mice prior to tumour-cell injection was able to reduce tumour growth to that seen in JAX mice. The authors concluded that this anticancer effect was mediated by augmented dendritic cell function [27].

### Metabolism

The metabolic activity of the microbiota can exert an oncosuppressive effect by a variety of mechanisms. SCFAs, including butyrate, acetate and propionate, are products of bacterial nondigestible carbohydrate fermentation that provide an important energy source for colonocytes and display anti-inflammatory, antiproliferative and pro-apoptotic properties. Butyrate is perhaps the best studied SCFA and induces NF-κB downregulation and T_reg_ cell induction [28]. Prebiotics can stimulate the growth of butyrogenic lactic acid bacteria*.* Prebiotic administration to rats increased the relative abundance of *Bifidobacteria*, increased SCFA production and afforded protection against CRC associated with chemically induced colitis [29]. However, the effects of pre-, pro- or synbiotic administration in humans has had mixed results with regards to increasing SCFA production. A 3-week diet of resistant starch (a prebiotic) was shown to increase faecal SCFA in one study [30], while in another, synbiotic administration in healthy human volunteers failed to demonstrate differences in faecal SCFA concentration or serum inflammatory markers [31]. Neither study was large (*n* = 17 at most) and firm conclusions cannot be drawn either way. A further mechanism by which probiotics may inhibit carcinogenesis is via detoxification of pro-/carcinogens. Some probiotic strains possess cell-surface peptidoglycans which have been shown to bind mutagens *in vitro* [4] but *in vivo* studies have failed to demonstrate a physiologically useful effect [32]. Certain probiotic strains have antioxidant properties which can inhibit carcinogenesis in rodent models [33]. The activities of a variety of gut-flora associated enzymes are implicated in carcinogenesis, ß-glucuronidase and nitroreductase, for example, because of their ability to generate carcinogens from compounds present in the gut lumen. Several human studies have demonstrated that probiotics can reduce the intestinal activity of these enzymes [34–36].

### Antiproliferative effects

The antiproliferative and pro-apoptotic properties of probiotic strains are well-documented. Lactic acid bacteria have been shown to induce or enhance apoptosis of cancer cell lines [37] and similar effects have been noted in murine models [38, 39]. Various mechanisms for these effects have been proposed. Modulation of cell signalling cascades (e.g. NF-κB and MAPK) can result in Tumour Necrosis Factor-α and caspase-dependent apoptosis while SCFAs, such as butyrate, can exert antiproliferative effects via histone deacetylase inhibition [40]. DNA damage is frequently both an effect and driver of uncontrolled cancer cell proliferation and probiotics display antigenotoxic properties in animals exposed to mutagens [41]. Unfortunately, few studies have investigated the effects of pre-, pro- or synbiotics on cellular proliferation in humans. Worthley *et al* [31] found that administration of a synbiotic to human volunteers did not affect ki67 expression (a marker of epithelial proliferation) or crypt cellularity in subsequent rectal biopsies. However, a well-conducted dietary swap study by O’Keefe *et al* did find that switching from a low-fibre, high-animal fat to a high-fibre (resistant starch), low animal fat diet reduced ki67 staining in intestinal mucosal biopsies [42]. Given the nature of the dietary change, it is not possible to ascribe these changes specifically to the fibre element but the results are certainly interesting.

## Cancer prevention

Given plausible mechanisms of anticancer activity, what evidence is there that pre, pro- and synbiotics can prevent the development of cancer in humans? In common with much microbiome-oncology science, the effects of probiotics on CRC is the most extensively researched paradigm due to its global frequency, the high density of the microbiota in the colon (orders of magnitude greater than any other host niche) and the significant impact of environmental (rather than genetic) risk factors. While there are no long-term prospective cohort studies investigating the effect of pre, pro- or synbiotic use on CRC incidence, large-scale epidemiological studies have examined the effects of yoghurt and unpasteurised milk intake, which typically contain live microorganisms. A prospective cohort study of 45,241 individuals showed that high versus low yoghurt intake was significantly associated with a decreased CRC risk (hazard ratio 0.65, 95% confidence interval (CI) 0.48–0.89) [43] while another trial of 41,836 participants found that unpasteurised milk consumption was associated with a lower risk of rectal (but not colon) cancer (relative risk (RR) 0.26, 95% CI 0.1-0.69) [44]. Fermented/cultured milk consumption has also been associated with a lower risk of bladder cancer [45, 46]. However, a meta-analysis demonstrated that milk and total dairy products were associated with a reduced risk of CRC (RR 0.81, 95% CI 0.74–0.90) but no significant association was demonstrated for either yoghurt or fermented dairy products alone [47]. As with many epidemiological studies of dietary factors, the evidence is suggestive but controversial and prospective data regarding pre-, pro and synbiotic use and on primary cancer risk specifically is lacking. A few studies have looked at their role in preventing recurrent cancers. In a randomised controlled trial (RCT) comparing synbiotic to placebo in polypectomised or CRC patients, Rafter *et al* [48] demonstrated the improvement in several CRC biomarkers, such as DNA damage and cellular proliferation, in the intervention group [48]. However, another investigation of the secondary prevention in patients with a prior history of CRC did not find that synbiotic administration prevented tumour recurrence [49]. A meta-analysis including nearly 11,000 subjects with colorectal adenomas concluded that high dietary fibre intake was associated a lower relative risk of subsequent progression to CRC (RR 0.72, 95% CI 0.63–0.83) [50]. Interestingly, prevention of recurrent bladder cancer (following transurethral resection) by oral *Lactobacillus casei Shirota* administration has proved more promising with two human trials reporting favourable results compared to placebo [49, 51].

## Cancer treatment

The use of bacterial agents in the treatment of cancer is not new: Bacillus Calmette–Guérin installation has been successfully used to treat superficial bladder cancer since 1977. Various murine models (alluded to above) have shown that probiotics can have direct anticancer effects and researchers are investigating how microbes may be genetically modified for enhanced anticancer efficacy or used as delivery vehicles for chemotherapeutics. However, the principal role of microbiome modulating therapy in human cancer is currently as an adjunctive therapy, optimising the efficacy and/or minimising the adverse effects of chemotherapy, radiotherapy, antibiotics and surgery.

### Chemotherapy and radiotherapy

The interaction between chemotherapeutic agents and the microbiome has been extensively reviewed elsewhere in this special issue (Pouncey *et al* [52]) but it is worth highlighting the specific areas where probiotics may be of benefit. A diverse microbial community is a key feature of the healthy intestinal microbiome and recent evidence suggests that it may also influence chemotherapeutic response and overall cancer survival. Murine models have highlighted the importance of the microbiome in the anticancer efficacy of chemotherapeutic agents [53, 54]. Low microbial diversity was independently associated with reduced survival in patients undergoing allogenic haematopoietic stem cell transplantation [55] and with a lack of clinical response to an immune checkpoint inhibitor in patients with advanced melanoma [56]. Furthermore, chemotherapy treatment itself has been shown to significantly reduce microbial diversity [57]. In this context, pre- and probiotic therapy could be considered prior to and along-side chemotherapy as a method to maintain diversity and promote chemotherapeutic efficacy. Studies addressing this question have not been published thus far but it is an interesting avenue for future investigation. Changes in bowel function are well-documented following pelvic radiotherapy, often delivered to patients undergoing treatment for urogynaecological or rectal malignancies. There is growing interest in the role of the microbiota in radiation-induced intestinal injury and the possible therapeutic use of probiotics [58]. Dysbiosis following pelvic radiotherapy has been associated with the development of post-radiotherapy diarrhoea [59] and a variety of randomised trials have demonstrated the ability of probiotics to attenuate radiation-induced GI side-effects [60]. Similarly, GI mucositis is a common adverse effect of many chemotherapeutic agents and can be dose limiting /treatment limiting. Probiotics may counter the pathophysiology of mucositis at multiple levels; activation of cyto-protective pathways, mitigation of reactive oxygen species, displacement of pathogenic bacteria and maintenance of intestinal barrier integrity. A randomised trial of probiotic supplementation versus placebo in 150 patients receiving 5-fluorouracil-based chemotherapy for CRC found significantly reduced grade 3/4 diarrhoea in the intervention group (22 versus 37%, *p* = 0.027) [61]. Meta-analysis has further shown that probiotic supplementation reduced the incidence of diarrhoea in cancer patients (odds ratio antibiotics (OR) 0.52, 95% CI 0.34–0.78) [62]. Expert guidelines have now been published advocating the use of *Lactobacillus*-containing probiotics to prevent diarrhoea in patients receiving chemotherapy or radiotherapy for pelvic malignancy [63].

### Antibiotics

A large proportion of cancer patients will take antibiotics at some point during their therapeutic journey which can have a rapid impact on microbiome composition and function [64]. For instance, the causative role of antibiotics in diarrhoea and *C. difficile* infection specifically is well-established. A Cochrane meta-analysis of 31 prospective randomised trials concluded that adjunctive probiotic administration can prevent *C. difficile* diarrhoea associated with antibiotic use (number needed to treat current status and safety 42 patients, 95% CI 32–58) [65]. Less well-appreciated is the detrimental effect antibiotic use may have on chemotherapeutic efficacy. Routy *et al* [66] recently demonstrated that antibiotic treatment for concomitant dental, pulmonary or urinary tract infections attenuated the clinical effect of immune checkpoint inhibitor therapy [66]. Antibiotic-treated patients had worse overall and progression-free survival. Follow-up studies in mice found that oral supplementation with *A. muciniphilia* could restore the clinical response to immune checkpoint inhibitor therapy in germ-free animals that had received faecal microbiota transplant from nonresponder patients [66]. This investigation provides further support for probiotic co-administration during antibiotic therapy though does raise the important question of which strains should be included in preparations.

### Perioperative prophylaxis

Resectional surgery is common for many oncology patients and there is significant evidence for a clinical benefit of pre-, pro- and synbiotics as perioperative adjuvant therapies. The majority of evidence concerns GI surgery in which localised and systemic infections and bowel dysfunction are common post-operative sequelae. Several meta-analyses of RCTs have been conducted; one of the largest, by Wu *et al* [67] in 2016, considered outcomes following elective oesophagogastric, colorectal and hepatobiliary surgery (one included paper concerned congenital heart disease surgery) in 34 trials with 2,634 participants [67]. Patients receiving perioperative syn- or probiotic therapy (in addition to standard perioperative care which includes antibiotic administration) had a significantly reduced risk of surgical site infection compared to those receiving a placebo (RR 0.65, 95% CI 0.51–0.84). Other positive effects included shortened length of antibiotic therapy, length of hospital stay, length of intensive care stay and earlier first bowel movement. Subgroup analyses of the infectious complications revealed significant reductions for pneumonia, wound infections, urinary tract infections and systemic sepsis but not intra-abdominal infections. Noninfectious complications, anastomotic leak and overall mortality were unaffected. Another analysis confined to patients undergoing colorectal resection demonstrated a reduction in post-operative infections (OR 0.39, 95% CI 0.22–0.68) but also reduced post-operative diarrhoea and symptomatic intestinal obstruction [68]. Three meta-analyses also compared the relative effectiveness of pre-, pro- and synbiotic therapies and all concluded that synbiotics were more effective than either pro- or pre-biotics alone in reducing post-operative infectious complications [67, 69, 70]. Importantly, no analysis demonstrated significant adverse events associated with treatment. As with many meta-analyses of microbiome modulating therapies, there was significant heterogeneity between the preparations administered (the majority containing *Lactobacillus spp.)* and no single preparation can be recommended with confidence. Few studies have shed mechanistic light on the observed effects though enhanced intestinal barrier function may be important [18]. Investigators have also examined the effectiveness of probiotics in managing post-operative bowel-related symptoms after resection for CRC. Following 3 months of treatment, patients receiving probiotics reported significantly improved bowel function and overall quality of life compared to patients receiving placebo [71].

## Current status and safety

The application of pre-, pro- and synbiotics to the prevention and treatment of cancer is an exciting and evolving space. These agents can be relatively cheaply produced, have significant beneficial effects and appear to have a very limited side-effect profile. Safety is of course of paramount concern in any patient population but cancer patients may be particularly vulnerable as they are often in a state of immunocompromise due either to the cancer itself or its treatment. However, a meta-analysis of 2,242 cancer patients receiving probiotic therapy to prevent GI toxicity did not demonstrate significant adverse events associated with treatment [62]. Despite this, there have been a handful of case-reports [72] of unusual infections in oncology patients taking probiotics though none were fatal. Similarly, such infections have also been noted in patients not known to be taking probiotics thus it is difficult to be certain of causality. Neutropenia is a common complication following chemotherapy and current recommendations by manufacturers are to avoid probiotics in these patients. However, there is no evidence that ingested probiotics pose any greater infective risk than those commensals already present in the GI tract. It also worth bearing in mind that even the highest colony-forming unit counts of probiotics rarely exceed 5 × 10 [10]; or approximately 0.1% of the total bacterial load in the average colon (4 × 10 [13]). A few small studies have investigated the safety of probiotic administration in neutropenic patients (reviewed by Mego *et al* [73]) without finding a significant increase in related infections. However, very rare events are unlikely to be picked up in such studies. Risks must be balanced against benefits and the possibility that probiotics may prevent or attenuate febrile neutropenia in oncology patients is being actively investigated (ClinicalTrials.gov Identifier NCT02544685). Certainly, trials in this patient cohort need to consider probiotics as they would any pharmaceutical intervention. It is also important to realise that the cat is rather out of the bag: a prospective survey of 500 oncology patients found that 29% were taking probiotics, approximately half of whom had no specific recommendation form their oncologist or pharmacist [74]. From the oncologists’ perspective, giving advice regarding pre-, pro- and synbiotics to patients does present some challenges. Specifically, it is difficult to advise patients on the preparation to take. There are a wide variety of preparations available over the counter, containing different species, strains and adjuncts at different concentrations and in different forms. There are rarely two studies on any given preparation and such heterogeneity is rarely taken into account in meta-analyses (if it was, such statistical summary wouldn’t be possible). There are some general guides available on probiotic products (e.g. Clinical Guide to Probiotic Products, 2017, available in the USA and Canada) that can be of use but, generally, it is up to the physician (or patient) to appraise the relevant literature and select a product that delivers the appropriate agent. Brand names are generally excluded from clinical guidelines as the focus should be on the specific strain or strain combination which has shown clinical benefit in a particular situation. To complicate matters further, it may well be that an individual’s basal microbiome influences their response to a given pre-, pro- or synbiotic – a fact rarely considered in trials.

## Conclusion

To conclude, pre-, pro- or synbiotics offer significant potential for the prevention and treatment of cancer but the field is very much in its infancy. There are well-evidenced mechanisms by which probiotics may exert beneficial effects but clinical studies are few in number, small, heterogenous and often suffer from significant biases [62]. With regards to cancer prevention, it is difficult to conclude that these agents have a benefit above that of a balanced diet—low in animal meat and high in fibre—but many Westerners do not follow such a diet and have other cancer risk factors, such as obesity and diabetes. The risks of taking pre- and probiotic supplements certainly appear to be minimal though the costs of long-term consumption would not be insignificant. Prospective longitudinal cohort studies are key to determine any benefit in terms of cancer risk. The use of pre-, pro- and synbiotics as adjuncts in the treatment of cancer has a better evidence base. In particular, there is moderate evidence to support their use in the prevention of antibiotic-associated diarrhoea, diarrhoea associated with chemotherapy or radiotherapy for pelvic malignancy and infectious complications following surgery. The potential benefits of co-administration with immunotherapy are just beginning to be appreciated and randomised interventional trials in humans are a key. However, for patients to derive the most benefit from probiotics we need to better understand the specific preparations that work in specific patient cohorts. It is an exciting time for pro- and prebiotic research, particularly within oncology, as many clinical benefits appear tantalisingly close. However, to realise them, there really needs to be a focus on conducting a good quality, well-reported trials.

## Conflicts of interest

Winclove Probiotics specialises in the research, development and manufacturing of probiotic products. AS and CM have solicited sponsorship from Winclove Probiotics to fund an educational meeting on the microbiome and cancer (ICMC 2017, London, UK) and have been guests at Winclove Probiotics’ expense. JY and EP are employees of Winclove Probiotics.

## References

[1] Gibson GR, Hutkins R, Sanders ME (2017). Expert consensus document: The International Scientific Association for Probiotics and Prebiotics (ISAPP) consensus statement on the definition and scope of prebiotics. Nat Rev Gastroenterol Hepatol.

[2] Hill C, Guarner F, Reid G (2014). Expert consensus document. The International Scientific Association for Probiotics and Prebiotics consensus statement on the scope and appropriate use of the term probiotic. Nat Rev Gastroenterol Hepatol.

[3] Oelschlaeger TA (2010). Mechanisms of probiotic actions—A review. Int J Med Microbiol.

[4] Orrhage K, Sillerstrom E, Gustafsson JA (1994). Binding of mutagenic heterocyclic amines by intestinal and lactic acid bacteria. Mutat Res.

[5] Jones ML, Tomaro-Duchesneau C, Prakash S (2014). The gut microbiome, probiotics, bile acids axis, and human health. Trends Microbiol.

[6] LeBlanc JG, Chain F, Martin R (2017). Beneficial effects on host energy metabolism of short-chain fatty acids and vitamins produced by commensal and probiotic bacteria. Microb Cell Fact.

[7] Commane D, Hughes R, Shortt C (2005). The potential mechanisms involved in the anti-carcinogenic action of probiotics. Mutat Res.

[8] Dimidi E, Christodoulides S, Scott SM (2017). Mechanisms of action of probiotics and the gastrointestinal microbiota on gut motility and constipation. Adv Nutr.

[9] Sanders ME (2011). Impact of probiotics on colonizing microbiota of the gut. J Clin Gastroenterol.

[10] Alexander J, Scott AJ, Pouncey AL (2018). Colorectal Carcinogenesis: an exemplar of gut microbiota–host interaction. Ecancermedicalscience.

[11] Thomas RM, Jobin C (2015). The microbiome and cancer: is the ‘oncobiome’ mirage real?. Trends Cancer.

[12] Schwabe RF, Jobin C (2013). The microbiome and cancer. Nat Rev Cancer.

[13] Dos Reis SA, da Conceicao LL, Siqueira NP (2017). Review of the mechanisms of probiotic actions in the prevention of colorectal cancer. Nutr Res.

[14] Caballero-Franco C, Keller K, De Simone C (2007). The VSL#3 probiotic formula induces mucin gene expression and secretion in colonic epithelial cells. Am J Physiol Gastrointest Liver Physiol.

[15] Anderson RC, Cookson AL, McNabb WC (2010). Lactobacillus plantarum MB452 enhances the function of the intestinal barrier by increasing the expression levels of genes involved in tight junction formation. BMC Microbiol.

[16] Resta-Lenert SC, Barrett KE (2009). Modulation of intestinal barrier properties by probiotics: role in reversing colitis. Ann N Y Acad Sci.

[17] Lewis MC, Merrifield CA, Berger B (2017). Early intervention with Bifidobacterium lactis NCC2818 modulates the host-microbe interface independent of the sustained changes induced by the neonatal environment. Sci Rep.

[18] Liu Z, Qin H, Yang Z (2011). Randomised clinical trial: the effects of perioperative probiotic treatment on barrier function and post-operative infectious complications in colorectal cancer surgery - a double-blind study. Aliment Pharmacol Ther.

[19] Karczewski J, Troost FJ, Konings I (2010). Regulation of human epithelial tight junction proteins by Lactobacillus plantarum in vivo and protective effects on the epithelial barrier. Am J Physiol Gastrointest Liver Physiol.

[20] Kuugbee ED, Shang X, Gamallat Y (2016). Structural change in microbiota by a probiotic cocktail enhances the gut barrier and reduces cancer via TLR2 signaling in a rat model of colon cancer. Dig Dis Sci.

[21] Fernandez NC, Lozier A, Flament C (1999). Dendritic cells directly trigger NK cell functions: cross-talk relevant in innate anti-tumor immune responses in vivo. Nat Med.

[22] Ogawa T, Asai Y, Tamai R (2006). Natural killer cell activities of synbiotic Lactobacillus casei ssp. casei in conjunction with dextran. Clin Exp Immunol.

[23] Gill HS, Rutherfurd KJ, Cross ML (2001). Enhancement of immunity in the elderly by dietary supplementation with the probiotic Bifidobacterium lactis HN019. Am J Clin Nutr.

[24] Nagao F, Nakayama M, Muto T (2000). Effects of a fermented milk drink containing Lactobacillus casei strain Shirota on the immune system in healthy human subjects. Biosci Biotechnol Biochem.

[25] Lim BK, Mahendran R, Lee YK (2002). Chemopreventive effect of Lactobacillus rhamnosus on growth of a subcutaneously implanted bladder cancer cell line in the mouse. Jpn J Cancer Res.

[26] Takagi A, Matsuzaki T, Sato M (2001). Enhancement of natural killer cytotoxicity delayed murine carcinogenesis by a probiotic microorganism. Carcinogenesis.

[27] Sivan A, Corrales L, Hubert N (2015). Commensal Bifidobacterium promotes antitumor immunity and facilitates anti-PD-L1 efficacy. Science.

[28] Vinolo MA, Rodrigues HG, Nachbar RT (2011). Regulation of inflammation by short chain fatty acids. Nutrients.

[29] Hu Y, Le Leu RK, Christophersen CT (2016). Manipulation of the gut microbiota using resistant starch is associated with protection against colitis-associated colorectal cancer in rats. Carcinogenesis.

[30] Phillips J, Muir JG, Birkett A (1995). Effect of resistant starch on fecal bulk and fermentation-dependent events in humans. Am J Clin Nutr.

[31] Worthley DL, Le Leu RK, Whitehall VL (2009). A human, double-blind, placebo-controlled, crossover trial of prebiotic, probiotic, and synbiotic supplementation: effects on luminal, inflammatory, epigenetic, and epithelial biomarkers of colorectal cancer. Am J Clin Nutr.

[32] Bolognani F, Rumney CJ, Rowland IR (1997). Influence of carcinogen binding by lactic acid-producing bacteria on tissue distribution and in vivo mutagenicity of dietary carcinogens. Food Chem Toxicol.

[33] Kumar RS, Kanmani P, Yuvaraj N (2012). Lactobacillus plantarum AS1 isolated from south Indian fermented food Kallappam suppress 1,2-dimethyl hydrazine (DMH)-induced colorectal cancer in male Wistar rats. Appl Biochem Biotechnol.

[34] Bouhnik Y, Flourie B, Andrieux C (1996). Effects of Bifidobacterium sp fermented milk ingested with or without inulin on colonic bifidobacteria and enzymatic activities in healthy humans. Eur J Clin Nutr.

[35] Ling WH, Hanninen O, Mykkanen H (1992). Colonization and fecal enzyme activities after oral Lactobacillus GG administration in elderly nursing home residents. Ann Nutr Metab.

[36] Marteau P, Pochart P, Flourie B (1990). Effect of chronic ingestion of a fermented dairy product containing Lactobacillus acidophilus and Bifidobacterium bifidum on metabolic activities of the colonic flora in humans. Am J Clin Nutr.

[37] Iyer C, Kosters A, Sethi G (2008). Probiotic Lactobacillus reuteri promotes TNF-induced apoptosis in human myeloid leukemia-derived cells by modulation of NF-kappaB and MAPK signalling. Cell Microbiol.

[38] Chen CC, Lin WC, Kong MS (2012). Oral inoculation of probiotics Lactobacillus acidophilus NCFM suppresses tumour growth both in segmental orthotopic colon cancer and extra-intestinal tissue. Br J Nutr.

[39] Le Leu RK, Brown IL, Hu Y (2005). A synbiotic combination of resistant starch and Bifidobacterium lactis facilitates apoptotic deletion of carcinogen-damaged cells in rat colon. J Nutr.

[40] Berni Canani R, Di Costanzo M, Leone L (2012). The epigenetic effects of butyrate: potential therapeutic implications for clinical practice. Clin Epigenetics.

[41] Pool-Zobel BL, Bertram B, Knoll M (1993). Antigenotoxic properties of lactic acid bacteria in vivo in the gastrointestinal tract of rats. Nutr Cancer.

[42] O’Keefe SJ, Li JV, Lahti L (2015). Fat, fibre and cancer risk in African Americans and rural Africans. Nat Commun.

[43] Pala V, Sieri S, Berrino F (2011). Yogurt consumption and risk of colorectal cancer in the Italian European prospective investigation into cancer and nutrition cohort. Int J Cancer.

[44] Sellers TA, Vierkant RA, Djeu J (2008). Unpasteurized milk consumption and subsequent risk of cancer. Cancer Causes Control.

[45] Ohashi Y, Nakai S, Tsukamoto T (2002). Habitual intake of lactic acid bacteria and risk reduction of bladder cancer. Urol Int.

[46] Larsson SC, Andersson SO, Johansson JE (2008). Cultured milk, yogurt, and dairy intake in relation to bladder cancer risk in a prospective study of Swedish women and men. Am J Clin Nutr.

[47] Aune D, Lau R, Chan DS (2012). Dairy products and colorectal cancer risk: a systematic review and meta-analysis of cohort studies. Ann Oncol.

[48] Rafter J, Bennett M, Caderni G (2007). Dietary synbiotics reduce cancer risk factors in polypectomized and colon cancer patients. Am J Clin Nutr.

[49] Ishikawa H, Akedo I, Otani T (2005). Randomized trial of dietary fiber and Lactobacillus casei administration for prevention of colorectal tumors. Int J Cancer.

[50] Ben Q, Sun Y, Chai R (2014). Dietary fiber intake reduces risk for colorectal adenoma: a meta-analysis. Gastroenterology.

[51] Aso Y, Akaza H, Kotake T (1995). Preventive effect of a Lactobacillus casei preparation on the recurrence of superficial bladder cancer in a double-blind trial. The BLP Study Group. Eur Urol.

[52] Pouncey A (2018). Gut microbiota, chemotherapy, and the host: the influence of the gut microbiota on cancer treatment. Ecancermedicalscience.

[53] Iida N, Dzutsev A, Stewart CA (2013). Commensal bacteria control cancer response to therapy by modulating the tumor microenvironment. Science.

[54] Viaud S, Saccheri F, Mignot G (2013). The intestinal microbiota modulates the anticancer immune effects of cyclophosphamide. Science.

[55] Taur Y, Jenq RR, Perales MA (2014). The effects of intestinal tract bacterial diversity on mortality following allogeneic hematopoietic stem cell transplantation. Blood.

[56] Gopalakrishnan V, Spencer CN, Nezi L (2018). Gut microbiome modulates response to anti-PD-1 immunotherapy in melanoma patients. Science.

[57] Montassier E, Gastinne T, Vangay P (2015). Chemotherapy-driven dysbiosis in the intestinal microbiome. Aliment Pharmacol Ther.

[58] Ferreira MR, Muls A, Dearnaley DP (2014). Microbiota and radiation-induced bowel toxicity: lessons from inflammatory bowel disease for the radiation oncologist. Lancet Oncol.

[59] Manichanh C, Varela E, Martinez C (2008). The gut microbiota predispose to the pathophysiology of acute postradiotherapy diarrhea. Am J Gastroenterol.

[60] Delia P, Sansotta G, Donato V (2007). Use of probiotics for prevention of radiation-induced diarrhea. Tumori.

[61] Osterlund P, Ruotsalainen T, Korpela R (2007). Lactobacillus supplementation for diarrhoea related to chemotherapy of colorectal cancer: a randomised study. Br J Cancer.

[62] Hassan H, Rompola M, Glaser AW (2018). Systematic review and meta-analysis investigating the efficacy and safety of probiotics in people with cancer. Support Care Cancer.

[63] Lalla RV, Bowen J, Barasch A (2014). MASCC/ISOO clinical practice guidelines for the management of mucositis secondary to cancer therapy. Cancer.

[64] Francino MP (2015). Antibiotics and the human gut microbiome: dysbioses and accumulation of resistances. Front Microbiol.

[65] Goldenberg JZ, Yap C, Lytvyn L (2017). Probiotics for the prevention of Clostridium difficile-associated diarrhea in adults and children. Cochrane Database Syst Rev.

[66] Routy B, Le Chatelier E, Derosa L (2018). Gut microbiome influences efficacy of PD-1-based immunotherapy against epithelial tumors. Science.

[67] Wu XD, Liu MM, Liang X (2018). Effects of perioperative supplementation with pro-/synbiotics on clinical outcomes in surgical patients: a meta-analysis with trial sequential analysis of randomized controlled trials. Clin Nutr.

[68] He D, Wang HY, Feng JY (2013). Use of pro-/synbiotics as prophylaxis in patients undergoing colorectal resection for cancer: a meta-analysis of randomized controlled trials. Clin Res Hepatol Gastroenterol.

[69] Kinross JM, Markar S, Karthikesalingam A (2013). A meta-analysis of probiotic and synbiotic use in elective surgery: does nutrition modulation of the gut microbiome improve clinical outcome?. JPEN J Parenter Enteral Nutr.

[70] Kasatpibal N, Whitney JD, Saokaew S (2017). Effectiveness of probiotic, prebiotic, and synbiotic therapies in reducing postoperative complications: a systematic review and network meta-analysis. Clin Infect Dis.

[71] Ohigashi S, Hoshino Y, Ohde S (2011). Functional outcome, quality of life, and efficacy of probiotics in postoperative patients with colorectal cancer. Surg Today.

[72] Cesaro S, Chinello P, Rossi L (2000). Saccharomyces cerevisiae fungemia in a neutropenic patient treated with Saccharomyces boulardii. Support Care Cancer.

[73] Mego M, Holec V, Drgona L (2013). Probiotic bacteria in cancer patients undergoing chemotherapy and radiation therapy. Complement Ther Med.

[74] Ciernikova S, Mego M, Semanova M (2017). Probiotic survey in cancer patients treated in the outpatient department in a comprehensive cancer center. Integr Cancer Ther.

[75] Velcich (2000). Colorectal cancer in mice genetically deficient in the mucin Muc2. Science.

